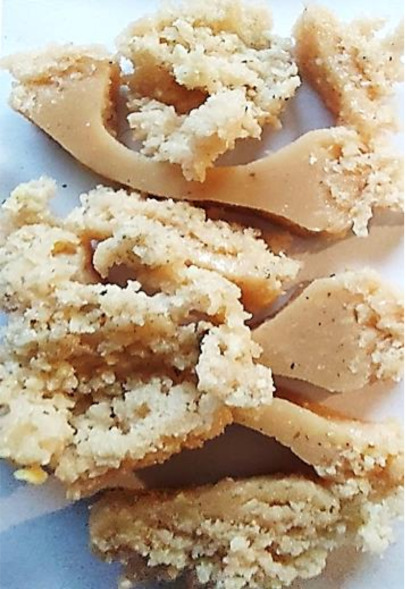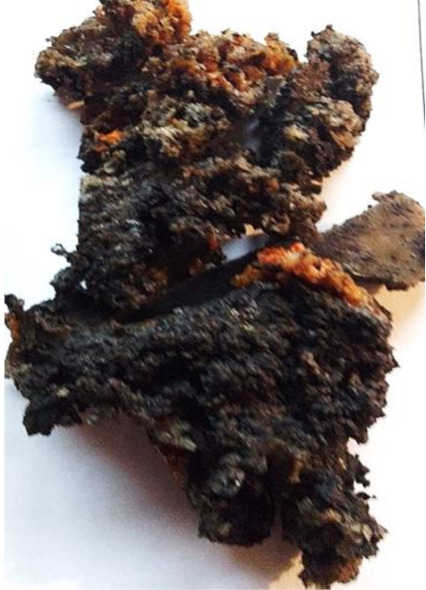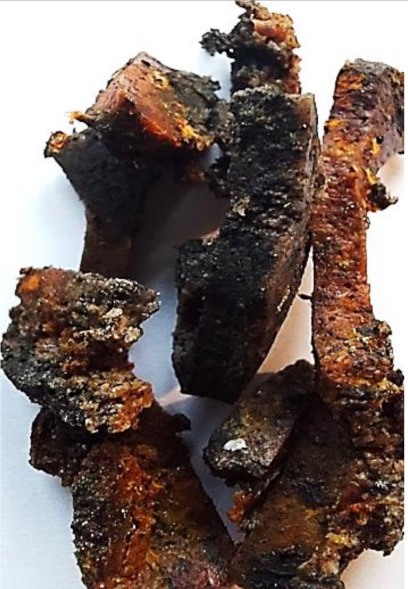# Evaluation of the Impact of Organic Fillers on Selected Properties of Organosilicon Polymer

**DOI:** 10.3390/polym13071103

**Published:** 2021-03-30

**Authors:** Sara Sarraj, Małgorzata Szymiczek, Tomasz Machoczek, Maciej Mrówka

**Affiliations:** Department of Theoretical and Applied Mechanics, Silesian University of Technology, Konarskiego 18A, 44-100 Gliwice, Poland; malgorzata.szymiczek@polsl.pl (M.S.); tomasz.machoczek@polsl.pl (T.M.); maciej.mrowka@polsl.pl (M.M.)

**Keywords:** organosilicon polymer, organic fillers, aging degradation, mechanical properties, eco-friendly materials

## Abstract

Eco-friendly composites are proposed to substitute commonly available polymers. Currently, wood–plastic composites and natural fiber-reinforced composites are gaining growing recognition in the industry, being mostly on the thermoplastic matrix. However, little data are available about the possibility of producing biocomposites on a silicone matrix. This study focused on assessing selected organic fillers’ impact (ground coffee waste (GCW), walnut shell (WS), brewers’ spent grains (BSG), pistachio shell (PS), and chestnut (CH)) on the physicochemical and mechanical properties of silicone-based materials. Density, hardness, rebound resilience, and static tensile strength of the obtained composites were tested, as well as the effect of accelerated aging under artificial seawater conditions. The results revealed changes in the material’s properties (minimal density changes, hardness variation, overall decreasing resilience, and decreased tensile strength properties). The aging test revealed certain bioactivities of the obtained composites. The degree of material degradation was assessed on the basis of the strength characteristics and visual observation. The investigation carried out indicated the impact of the filler’s type, chemical composition, and grain size on the obtained materials’ properties and shed light on the possibility of acquiring ecological silicone-based materials.

## 1. Introduction

Concern for the natural environment has forced scientists to intensify their efforts to develop new materials that will combine the required operational properties with the optimal ones in terms of the negative impact on the surroundings, as well as occupational health and safety. Hence, in recent years, extensive research has been conducted on composites with polymer matrices modified with organic fillers. Natural fiber-reinforced composites (NFRCs) allow obtaining degradable, eco-friendly alternatives for nonbiodegradable polymeric materials, which reduces the problem of costly waste disposal. Widely known wood–plastic composites (WPCs), owing to their superior properties (lightweight reasonable strength and stiffness), low production cost, potential recyclability, and high availability, have been burgeoning rapidly in recent years, with North America and China being the two largest producers and Europe being the third [1,2]. Previous research proved that organic fibers used as fillers improve mechanical properties, increase heat resistance, and reduce shrinkage and the tendency to cracking compared to pure polymeric materials [3,4,5]. The biocomposites described in the literature are most often produced on a matrix of thermoplastic polymer materials, such as polyethylene or polypropylene. This type of composites can be obtained using various technologies, among them extrusion or injection. Thanks to their operational characteristics, they have found increasing application in various industries, e.g., construction (terraces, swimming pool surroundings, floors, and facades), automotive (door panels, insulation, spoilers), or furniture industries (chairs, shelves, and benches) [5,6,7]. A new approach in obtaining biodegradable composites is the introduction of fillers, such as shells or coproduction wastes. It was proved that the incorporation of sunflower husk, hazelnut shells, and walnut shells led to a decrease in flammability and showed improved stiffness and hardness compared to unmodified epoxy resin [5,8]. In a similar study by Barczewski et al. [3], the obtained results showed that incorporating sunflower husk into ultra-low-density polyethylene improved the tensile strength and elastic modulus of the acquired material. Scientists [4,9] have studied the influence of introducing pistachio shells on the mechanical properties of composites on a polyester matrix. The obtained results indicated an increase in flexural strength and hardness of the prepared composites at 10 wt%. Nonetheless, similar studies conducted on epoxy polymers have shown that the addition of powdered pistachio shells, depending on particle size, improved or worsened the mechanical properties [10]. In a study on the effect of introducing spent coffee waste into polypropylene, the results showed a decrease in the mechanical properties of the composite due to the high fat content and granular porous form of the wastes [11]. This indicates the importance of particle size and form and their effect on the resulting material properties. However, it is rather difficult to find studies on synthetic organosilicon polymers modified with organic fillers obtained from plants’ waste in the literature. So far, the field of application of silicone composites has included thermal protection systems in which ceramifiable silicone rubber composites are used to increase the flame retardancy of electrical cables [12,13], the production of white light by photometric and colorimetric conversions in light-emitting diode (LED) packages [14,15], and various marine antifouling surfaces [16,17,18]. Silicone-based composites can provide elastic technical materials, and by using appropriate fillers, composites with highly desired properties can be produced. Beter et al. [19] investigated the influence of reinforcing silicone with glass fiber. The study revealed that the fibers increased stiffness and provided the possibility of developing tailored, flexible materials. In a study by Mrówka et al. [20] on the impact of wood waste on silicone-based composites, it was proved that WPC on a silicone matrix showed a relatively good biological response to aging in artificial seawater conditions. Simultaneously, the composites maintained high mechanical properties depending on the type of tree waste. In another study, researchers investigated the effect of zinc waste products on silicone-based composites’ tribological properties. The results showed that the 5% addition of zinc ash reduced the composites’ abrasion by 70% compared to the unmodified silicone [21]. Therefore, it seems that organosilicon polymers’ specific properties will allow the application area to be extended to cover materials such as coatings that could constitute a bioactive substrate, which the authors took upon investigating.

This study’s objective was to assess the impact of different organic fillers (ground coffee waste, walnut shell, brewers’ spent grains, pistachio shell, and chestnut) on the physicochemical and mechanical properties of silicone composites used as coating materials for structural elements. The studied composites contained 10 and 20 wt% of the mentioned fillers. The comparative evaluation was conducted by means of several physicochemical and mechanical tests (i.e., density, hardness, rebound resilience, abrasiveness, and static tensile test). The prepared composites were assessed for the degree of degradation in the artificial seawater environment. This is due to the potential application to environmental protective coatings.

## 2. Materials and Methods

### 2.1. Material Preparation

In this paper, food-grade addition-crosslinking silicone RTV22F was used as the polymer matrix (Table 1).

Five organic fillers (ground coffee waste, walnut shell, brewers’ spent grains, pistachio shell, and chestnut) were selected to acquire the composites. The chemical composition of the fillers is presented in Table 2.

In the chestnut case, raw nuts were roasted in an oven at 200 °C for 30 min. Before the heat treatment, the chestnut shell was pierced with incisions at an angle of 45°, which allowed for the pulp’s acquisition. The fillers were ground using a two-blade grinder with a crushing capacity of 150 g/min to obtain fine and homogenous grains. The next step was drying the fillers at a temperature of 60 °C for 24 h.

**Figure 1:**
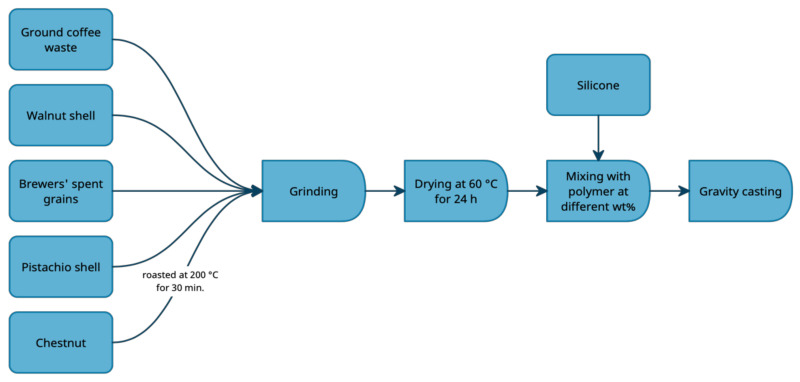
Material preparation scheme.

For silicone preparation, the base and the catalyst were mixed following the manufacturer’s instructions (mass ratio of 1:1). Upon the drying process, ten series of composites with the content of the fillers equaling 10 and 20 wt%, as well as the reference material, were cast into forms sized 150 mm × 200 mm × 4 mm. The samples were cured at room temperature for one day. Due to the desired porosity of the acquired material, vacuum deaeration was not performed. The material preparation scheme is shown in Figure 1. Dosing of the components was carried out using a lab balance (precision: ±0.001 g). The list of the obtained materials with the fillers’ concentration is presented in Table 3.

### 2.2. Research Methodology

#### 2.2.1. Scanning Electron Microscopy (SEM) Analysis

The morphology of the fillers was visualized using a Zeiss Supra 35 scanning electron microscope (Carl Zeiss AG, Oberkochen, Baden-Württemberg, Germany). Prior to the tests, each type of filler was sputtered with gold powder for 60 s. The electron accelerating voltage of 3 kV was applied, and magnifications of 500× and 5000× were used.

#### 2.2.2. Particle Size Analysis

The particle size distribution characterization was assessed using a Fritsch Analysette 22 Micro Tec Plus laser particle sizer (FRITSCH GmbH, Idar-Oberstein, Rhineland-Palatinate, Germany) equipped with a wet dispersing unit. The operation range was 0.08–2000 μm.

#### 2.2.3. Density

The density test of the prepared composites was carried out using an analytical balance (Ohaus Adventurer Pro, OHAUS Europe GmbH, Nänikon, Greifensee, Switzerland) equipped with a hydrostatic density measurement kit. It was realized following the standard ISO 1183-1 [27]. The test consisted of measuring the sample’s weight in air then in distilled water with known density (ρ = 0.998 g/cm3). From the obtained results, the density ρ (g/cm3) of the samples was determined according to Equation (1):

(1) $$\rho =\;\rho_{w}\frac{m_{1}}{m_{1}-m_{2}},$$

where $m_{1}$(g) and $m_{2}$(g) are the mass of the sample in air and distilled water, respectively, and $\rho_{w}$ (g/cm3) is the density of distilled water. The measurements were repeated five times for each material.

#### 2.2.4. Hardness

The hardness measurements were performed with the use of a Shore type A durometer (Zorn Stendal, Saxony-Anhalt, Germany) and were realized according to the standard ISO 7619-1 [28]. The study consisted of five measurements at a distance of at least 10 mm from the sample’s edge.

#### 2.2.5. Rebound Resilience

The rebound resilience test was realized using the Schob machine (Heckert, Chemnitz, Germany) to evaluate the elasticity of the prepared composites. The samples measured 30 mm × 30 mm × 4 mm. Prior to tests, samples were mechanically conditioned with two impacts. The measurements consisted of hitting the samples with a weight placed on a pendulum and reading off the value indicated by the pointer (%). Each sample was tested three times in accordance with the standard ISO 4662 [29].

#### 2.2.6. Tensile Testing

**Figure 2:**
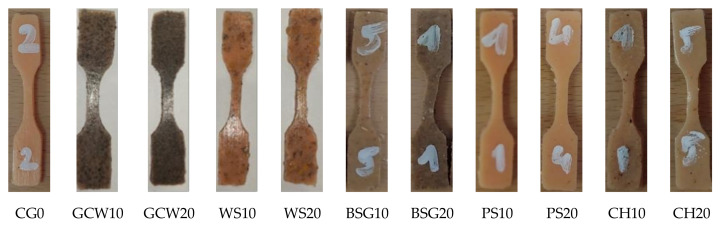
Prepared composites.

The static tensile test was carried out using an MTS Insight 10 testing machine (MTS Systems Corporation, Eden Prairie, Minn., USA) designed for uniaxial static excitation. The samples of all materials were 60 mm long and 4 mm wide along the measuring length; however, they differed in thickness, which resulted from the specificity of the used fillers (high hydrophilic properties). The crosshead speed was 50 mm/min. The samples were installed using pneumatic grips supplied with a pressure of 0.5 atmosphere (a value determined experimentally, which did not cause permanent damage to the tested samples), ensuring secure fixation. Due to the high mechanical flexibility of the specimens noticeable during assembly in the grips’ jaws, it was decided to start the tests after the stabilization of the force caused by the relaxation of the material. The measurement of the load in the displacement domain started with the increase of the reaction force generated by individual samples subjected to tension. The test was carried out until the destruction of the sample, indicated by the separation of the material. The maximum force values as a function of displacement were used for the comparative analysis. The test was performed 30 days after casting on five samples for each variant of material. Specimens for the static tensile test were cut according to the dimensions required by the standard ISO 527-1 (type 5-B) (Figure 2) [30].

#### 2.2.7. Accelerated Aging

The composites were subjected to an accelerated aging process by immersing them in artificial seawater conditions and kept at 70 °C. The samples had the shape adopted for tensile testing. Artificial seawater was prepared according to the standard ASTM D 1141-52 (Table 4) [31]. The aging time was 7 and 28 days. The degradation degree was assessed on the basis of a comparison of changes in strength characteristics after aging with respect to the unaged reference samples.

All tests were carried out at a temperature of 20 ± 2 °C and 50% humidity.

## 3. Results and Discussion

### 3.1. Natural Fillers Characterization

**Figure 3:**
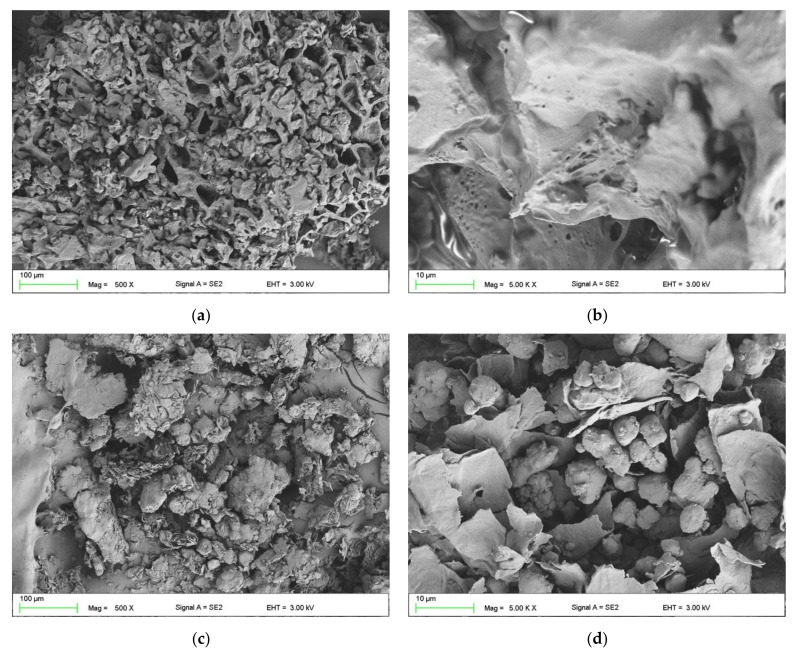

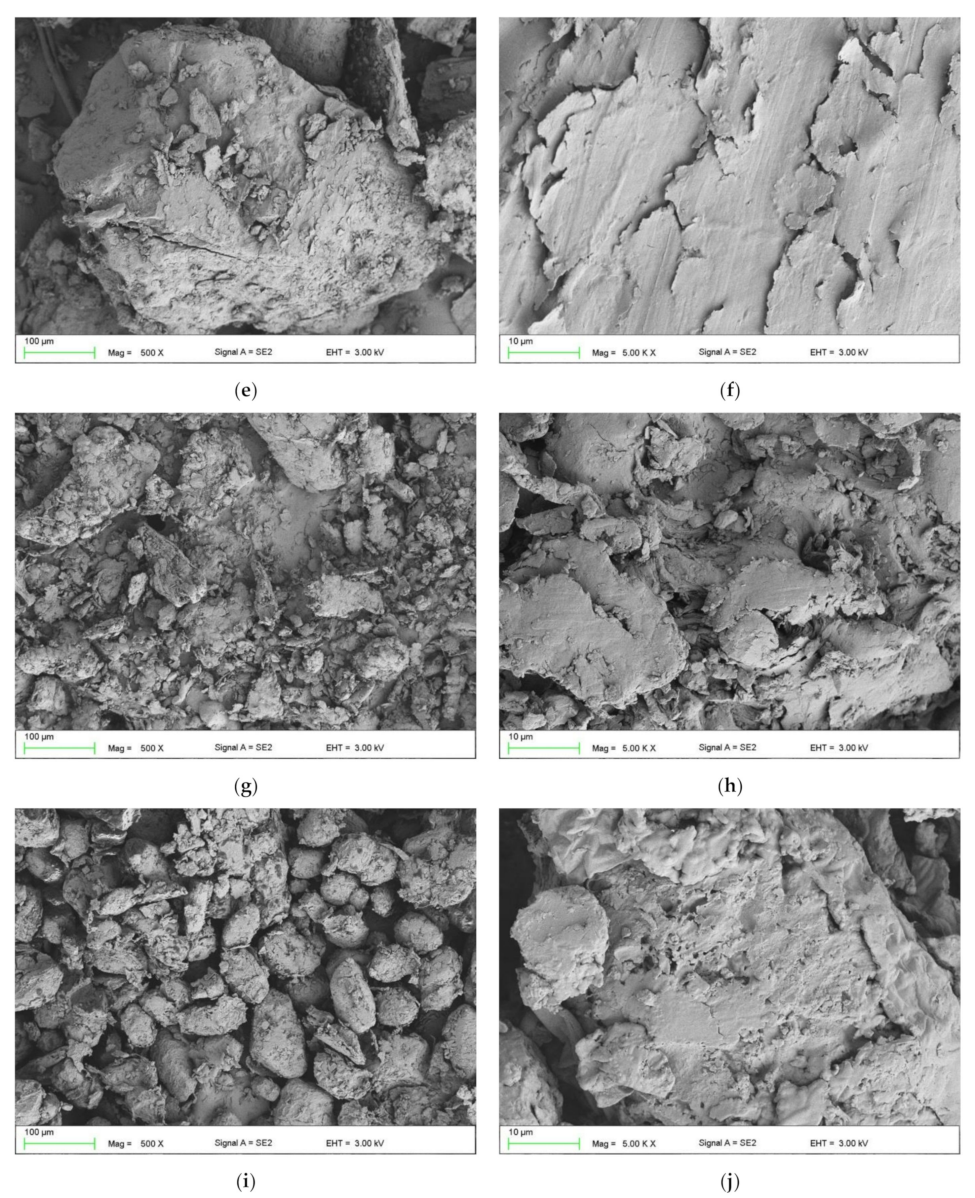
SEM images of ground coffee waste (GCW) (**a**,**b**), walnut shell (WS) (**c**,**d**), brewer’s spent grains (BSG) (**e**,**f**), pistachio shell (PS) (**g**,**h**), and chestnut (CH) (**i**,**j**).

The SEM images (Figure 3) confirmed that the obtained particles differed in size and shape. The most significant difference in the appearance was observed between ground coffee waste and the remaining fillers. In walnut shell (WS), brewer’s spent grains (BSG), pistachio shell (PS), and chestnut (CH) particles, irregular-shaped granules were predominant (Figure 3c,e,g,i), as well as greater surface roughness, whereas the ground coffee waste (GCW) particles resembled a sponge-like net of grains with visible pores (Figure 3a,b). This could be due to the dissolution of some ground coffee components during brewing. Although high surface roughness may adversely affect the wetting of fillers by the polymer, the particles’ well-developed surface increases the contact surface between the matrix and the filler, contributing to better mechanical adhesion.

**Figure 4:**
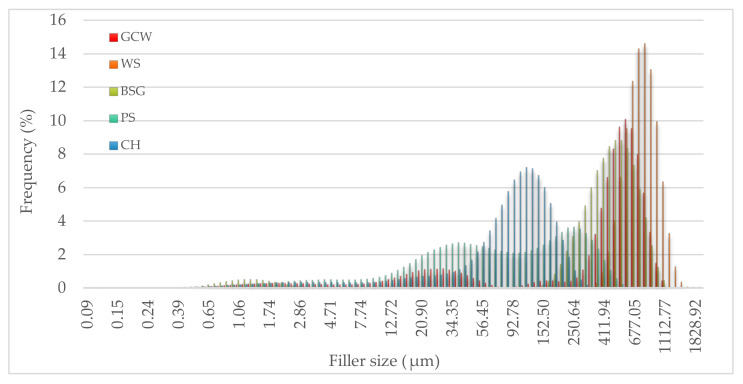
Frequency curves of the fillers.

**Figure 5:**
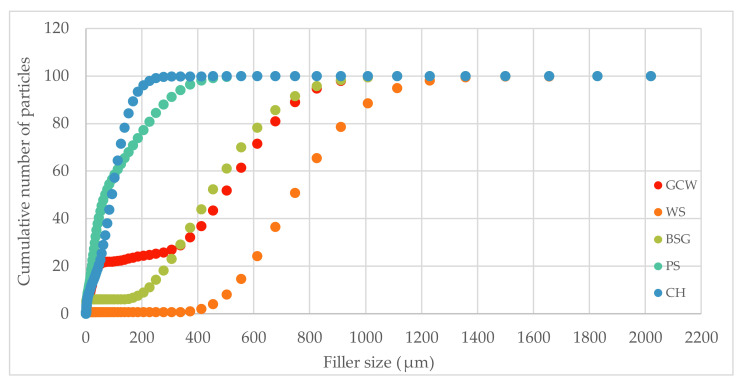
Cumulative particle number of the fillers.

The frequency curves and the cumulative particle number of the fillers were based on five measurements for each type of material (Figure 4 and Figure 5).

The most frequent particle size of the fillers differed tremendously, with CH having the smallest most common size at 113.18 μm (7.2%) and WS having the biggest at 825.91 μm (14.6%). BSG and GCW had relatively similar common sizes, 555.02 (8.8%) and 613 μm (10.1%), respectively. In the case of PS, two frequent particle sizes were observed, 37.94 (2.7%) and 250.64 μm (3.7%).

As shown in Figure 5, 90% of PS and CH particles had the smallest size, 295.92 μm and 171.59 μm, respectively. BSG (728.92 μm) and GCW (760.59 μm) had comparable particle sizes. The biggest particles were observed for WS having the size 1032.11 μm. The noticeable difference could be due to the fillers’ different structure morphology and their individual behavior during the grinding process.

### 3.2. Density Test Results

**Figure 6:**
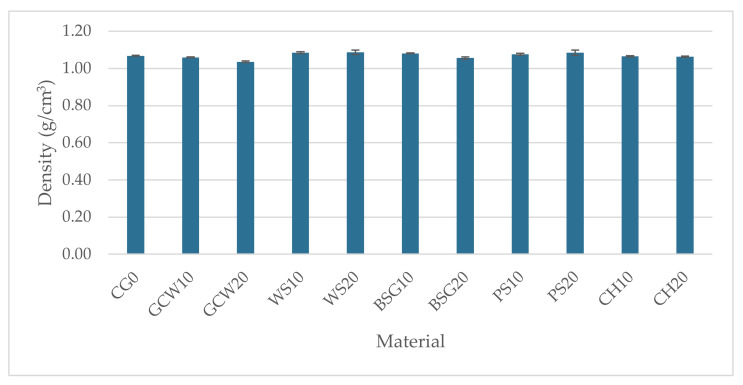
Materials’ density (g/cm^3^).

The average density of the obtained composites, along with the standard deviation, is graphically illustrated in Figure 6.

The control group samples showed an average density of 1.07 g/cm^3^, while the density specified by the producer was 1.1 g/cm^3^. The difference is 2.72%, which could probably result from air bubbles enclosed in the material structure. Deaeration of the composition would minimize the difference. In terms of the effect of fillers percentage addition on the composites’ density, no unequivocal trend was observed, and the density ranged between 1.04 and 1.09 g/cm^3^. For composites filled with 10 wt%, the ground coffee waste-silicone composite (GCW10) density was the lowest (1.06 g/cm^3^). In contrast, it increased for the walnut shell-silicone composite (WS10) by approximately 1.5% in comparison with the unmodified material. The 20 wt% filled composites varied the most, with the density of GCW20 (1.04 g/cm^3^) decreasing by approx. 3% compared to the control group samples, while for WS20 (1.09 g/cm^3^), it increased by approx. 2%. The overall percentage difference between the highest and the lowest density equaled 4.6%. The highest densities were observed for walnut shell-silicone composites at different mass weight ratios. This could be due to the structure, size, and weight of the particles, hence the filler’s sedimentation. The low density of ground coffee waste-silicone composites, both for the filler mass weight of 10% and 20%, could mainly result from the particles’ porous structure (Figure 3a), which allowed an even distribution of the filler in the polymer matrix. The statistical analysis proved an influence of the filler type and its content on the obtained materials’ density (F = 28.22, F crit = 2.05).

### 3.3. Hardness Test Results

**Figure 7:**
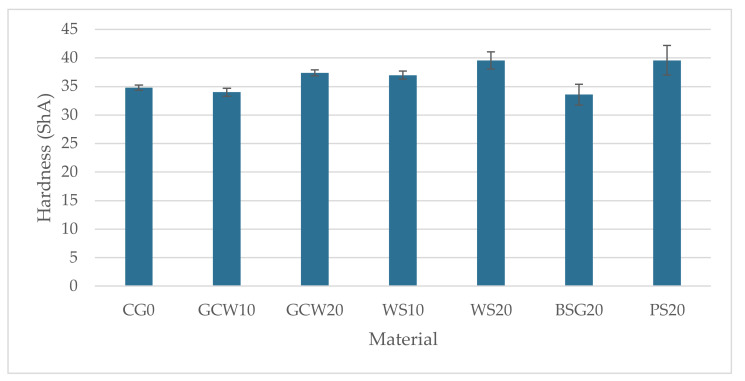
Materials’ hardness (ShA).

The average hardness of the obtained materials, along with the standard deviation, is graphically illustrated in Figure 7.

BSG10, PS10, CH10, and CH20 hardness were not included in the bar graph due to the low value, which was off the Shore A hardness scale. This could result from encountering air bubbles as the composites were not deaerated after casting or the sedimentation of the fillers. Much like the density results, no unequivocal trend was observed for hardness values. The highest hardness was recorded for WS20 and PS20, 39.6 ShA (approx. 14% higher than the reference material), which may be caused by the high cellulose and lignin content that provides strength to the shells [10,24]. The lowest hardness was noted for BSG20 (33.6 ShA), and it decreased by approx. 4% compared to the unmodified material and 18% compared to the highest registered hardness (WS20 and PS20). The conducted one-way variance analysis showed an influence of the filler type and content on the materials’ hardness values (F = 15.86, F crit = 2.45).

### 3.4. Rebound Resilience Test Results

**Figure 8:**
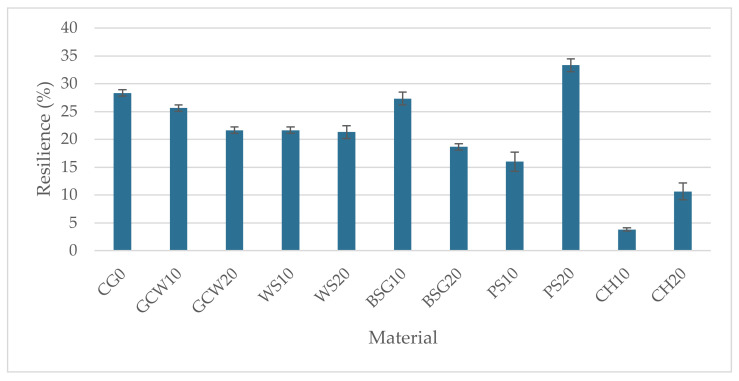
Materials’ rebound resilience (%).

The average resilience of the obtained materials, along with the standard deviation, is graphically illustrated in Figure 8.

The introduction of organic fillers influenced the resilience of obtained composites to varying degrees. The resilience of the PS20 composite was the highest (33.33%), and it increased by approx. 18% compared to the unmodified material. This may be related to the size and the shape of the filler particles. The lowest resilience was recorded for the CH10 composite at 3.83% (decreased by 86%). This could be caused by high starch content in roasted chestnut (65 g/100 g of dry mass) [32], which resulted in increased water absorption affecting the mechanical strength of the composite. For ground coffee waste, walnut shell, and brewers’ spent grains composites, the introduction of higher mass weight filler content reduced the materials’ resilience. The opposite is observed for pistachio shell and chestnut composites. The introduction of 20% pistachio shell increased resilience by 108% compared to the 10% filler content samples. For chestnut-silicone composites, the resilience of samples with 20 wt% of the filler increased by 178% compared to the ones with lower filler content. It can be concluded that, apart from the PS20 composite, the introduction of organic fillers at different mass weight ratios decreased the resilience of the obtained materials. The statistical analysis showed a significant impact of the filler type and its content on the obtained materials’ resilience (F = 208.46, F crit = 2.3).

### 3.5. Tensile Testing Results

**Figure 9:**
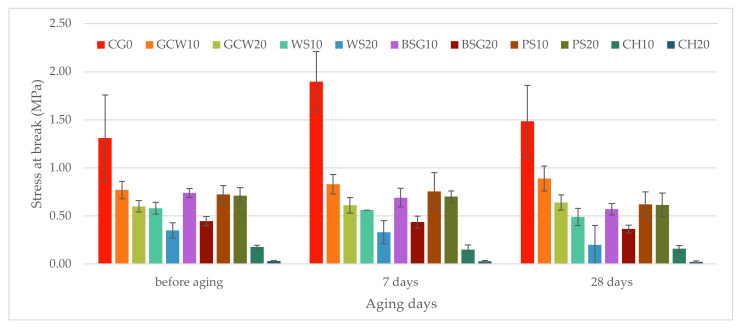
Materials’ stress at break (MPa).

**Figure 10:**
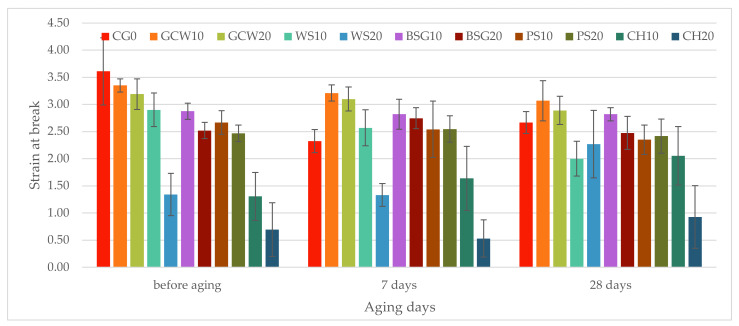
Materials’ strain at break.

The static tensile strength test was performed on native samples as well as the aged ones. Graphical representation of the obtained results with the standard deviation is presented for stress at break in Figure 9 and strain at break in Figure 10.

The highest stress at break was recorded for the reference material (1.23 MPa). The addition of any filler in both 10% and 20% filler content reduced the stress value. Moreover, the higher the filler content, the bigger decrease of stress at break value was recorded. The lowest values were observed for chestnut-silicone composites at 10% (0.18 MPa) and 20% (0.03 MPa) filler fraction, where the stress was reduced by approximately 86% and 97%, respectively. The highest stress values for the obtained composites were recorded for GCW10 (0.77 MPa) and BSG20 (0.74 MPa). This could be due to similar lignin content (responsible for plants’ rigidity) [23,25]. The percentage difference between the highest recorded stress value for composites (GCW10) and the lowest (CH20) equaled 96%. Only in the case of pistachio shell-silicone composites were the noted values comparable (10 wt%, 0.72 MPa; 20 wt%, 0.71 MPa). The relatively high stress at break value could be due to the small size of pistachio shell particles, which allowed an even distribution of the filler throughout the polymer matrix. On the contrary, the drop of stress value for the rest of the materials with the introduction of a higher filler content ranged from 23% for ground coffee waste-silicone composites up to 81% for chestnut-silicone composites. ANOVA analysis showed an impact of the studied factors on the stress value (F = 30.49, F crit = 2.07).

Aging in artificial seawater altered the tensile strength properties of the materials. For the unmodified material, stress at break increased after aging for 7 days (1.90 MPa, a 45% increase compared to the native sample) and slightly dropped to 1.48 MPa (13% increase) after 28 days. This could be caused by increased crystallinity of the polymer after aging [33]. Only in the case of GCW composites, for 10% and 20% filler fractions, stress increased with aging days (8% and 16% after 7 days and 2% and 7% after 28 days compared to the native samples). WS and BSG composites for both filler contents, as well as PS20, had a similar behavior, i.e., decreasing stress at break with increasing aging days in relation to the values before the aging process. The PS10 composite, much like GC0 samples, showed an increase of stress value after aging for 7 days and a drop after 28 days. In the case of chestnut-silicone composites, for both filler fractions, stress at break values maintained a comparable level after aging.

The reference material samples showed the greatest strain at break (3.61), which is relatively similar to what the producer indicated. As can be seen, the addition of organic fillers reduced that value to varying degrees. Furthermore, the higher the filler content, the bigger decrease of strain at break value recorded. The biggest strain at break among the obtained composites was observed for GCW10 and GCW20 (3.35 and 3.19, respectively). The highest value decrease was observed for chestnut composites (10 wt%, 1.3; 20 wt%, 0.69), which is correlated with the reduced rebound resilience of these materials. Moreover, for these composites, a high standard deviation was recorded, which could be due to the filler and materials’ preparation technique, resulting in the differentiation of the obtained specimens. Within the 10% composites, the difference between GCW10 and CH10 was the biggest, with a drop of strain value equaling 61%. Like the case of the 10% composites, for the 20% filled materials, the biggest difference was recorded for the same fillers equaling 78%. The most significant difference for the two filler fractions was between walnut shell-silicone composites equaling 54%. It can be concluded that the type of the filler and its content play a significant role in the obtained materials’ mechanical properties, which was confirmed by the carried out statistical analysis (F = 36.04, F crit = 2.07).

The conducted aging process affected the materials’ strain at break in varying ways. After aging, the greatest values were observed for ground coffee waste-silicone composites equaling 3.21 for 10% filler content and 3.10 for 20% after 7 days. The strain decreased after aging for 28 days (3.07 and 2.89, respectively); however, it remained the greatest recorded values for that aging period. For the unmodified material, a drop of value was recorded. The same trend was observed for the obtained composites aside from WS20, BSG20, PS20, CH10, and CH20. WS20 and CH20 had similar behavior, where the strain value decreased after aging for 7 days (1% and 23%, respectively) to increase again after aging for 28 days (69% and 34%, respectively). For BSG20 and PS20, strain at break increased after one week of aging (9% and 3%, respectively) and decreased after four weeks (2% for both composites). Only in the case of CH10, the more extended period of aging, the higher value of strain was recorded, increasing by 25% after 7 days and by 57% after 28 days.

### 3.6. Accelerated Aging Results

Aging effects in artificial seawater conditions on the obtained materials are presented in Table 5. It should be noted that the pictures were taken after conducting the tensile strength test.

Apart from the impact on the material’s tensile strength (Section 3.5), accelerated aging under artificial seawater conditions did not affect the structure or the organoleptic properties of the unmodified silicone. However, it did alter the properties of the obtained composites to a greater or lesser extent depending on the filler’s size and fraction. In the case of GCW composites, the coffee scent was intense before aging and decreased after seven days in artificial seawater. Moreover, the composites start to crumble, and changes in the samples’ color and swelling are observed. For walnut shell composites, the filler’s sedimentation is observed, which is related to the particle size. The color of the composites changes after aging for 7 and 28 days. The effect is more intense in the case of composites with a 20% filler fraction. Furthermore, surface defects were detected due to the sediment layer causing stresses leading to deformation of the material. However, this effect is not as significant as in the case of pistachio shell composites. For BSG composites, swelling is observed (the greatest with respect to the unmodified material, with up to a 58% difference in thickness), as well as changes in smell and color. PS samples deformed as a result of stress. Sedimentation of the filler and changes in its color from cream to brown, as well as the thickness alteration of the samples (17% increase), are observed. Chestnut composites changed color from bright beige to dark brown and black. The samples became spongy, and mold on their surface was observed. Solely in CH composites, the odor altered to a very unpleasant one (similar to bad composting of organic garden waste).

## 4. Conclusions

The results achieved in the research carried out provided further insight on the potential applications of eco-friendly silicone composites and allowed the following conclusions to be reached:

The introduction of organic fillers altered the composites’ density. A reduction of density by approximately 3% for GCW20 and an increase up to 1.5% for WS10 was observed (Figure 6).

The hardness of the obtained composites differed depending on the filler type and its content (Figure 7). The introduction of fillers at 20 wt% improved the hardness of the materials with the exception of the chestnut-silicone composite, for which hardness could not be measured due to the off-scale value. Only in the case of walnut shell-silicone composites at both filler fractions was an increase of the hardness value observed. However, based on the conducted statistical analysis, the filler type and its content are of minor importance for the tested characteristic.

The introduction of the studied fillers changed the resilience of the composites. A reduced resilience value was recorded for all composites apart from PS20, where it increased by approximately 18% compared to the reference material (Figure 8). A strong dependency of the filler type and mass weight on the tested characteristic was observed.

The tensile testing results showed reduced stress at break and strain at break values for the obtained composites at 10% and 20% filler content (Figure 9 and Figure 10). This decrease in most cases corresponded to the reduced value of resilience of the materials. Moreover, the lowest reduction of stress and strain values was observed for GCW10 (37% and 7%, respectively), whereas the highest reduction was recorded for CH20 (97% and 81%, respectively). This is mainly due to the chemical composition of the fillers (Table 2).

Accelerated aging altered the mechanical and organoleptic properties of the materials. For GCW composites, stress at break value increased with the aging period, and the highest strain at break values was recorded for these materials. The lowest values for tensile strength properties were recorded for CH composites. The significant changes in appearance were observed in the case of chestnut-silicone composites (changing of color and formation of mold).

It can be concluded that changes occurring in the material are highly dependent upon the filler’s chemical composition, structure, and grain size. Although the organic fillers altered to varying degrees the mechanical properties of the obtained composites, these changes are of little importance in terms of creating an eco-friendly bioactive substrate. The acquired materials can be used as a substrate suitable for the growth of living organisms, especially in the marine environment, and as a protection of wooden or steel marine structures, making them more compatible with the seabed. Moreover, such composites could have potential applications as packaging materials due to their degradability and lack of negative impact on the environment.

## Figures and Tables

**Table 1:** Properties of RTV22F silicone [22].

Property	Unit	Value
Density	(g/cm^3^)	1.1
Viscosity	(mPa⋅s)	3250
Hardness	(ShA)	22
Tensile strength	(MPa)	3
Tensile strain	(%)	350

**Table 2:** Chemical composition of the fillers [10,23,24,25,26].

Chemical Components	Composition (g/100 g Dry Material)				
	Ground Coffee Waste	Walnut Shell	Brewers’ Spent Grains	Pistachio Shell	Chestnut
Cellulose	12.4	23.9	17	42	3.58
Hemicellulose	39.1	22.4	27	-	-
Lignin	23.9	50.3	28	16.61	-
Protein	17.44	-	-	-	4.88
Ash	1.3	3.4	-	1.26	1.02
Fat	2.3	-	-	-	0.49
Polysaccharides	-	-	28	-	75.32

**Table 3:** Mass concentration of the organic fillers.

Filler	Content (%)	Material Code
Control Group	–	CG0
Ground Coffee Waste	10	GCW10
	20	GCW20
Walnut Shell	10	WS10
	20	WS20
Brewers’ Spent Grains	10	BSG10
	20	BSG20
Pistachio shell	10	PS10
	20	PS20
Chestnut	10	CH10
	20	CH20

**Table 4:** Chemical composition of artificial seawater [31].

Ingredients	Concentration (g/L)
Sodium chloride (NaCl)	24.53
Magnesium chloride (MgCl_2_)	5.2
Sodium sulfate (Na_2_SO_4_)	4
Calcium chloride (CaCl_2_)	1.16
Potassium chloride (KCl)	0.695
Sodium bicarbonate (NaHCO_3_)	0.201
Potassium bromide (KBr)	0.101
Boric acid (H_3_BO_3_)	0.027
Strontium chloride (SrCl_2_)	0.025
Sodium fluoride (NaF)	0.003

**Table 5:** Accelerated aging effect on the obtained materials.

Material	Aging Days		
	Before Aging	7 Days	28 Days
CG0	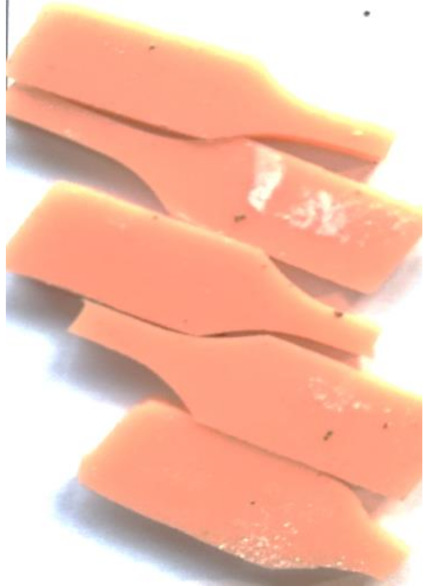	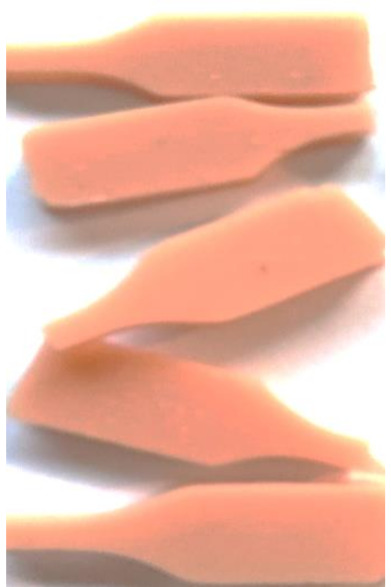	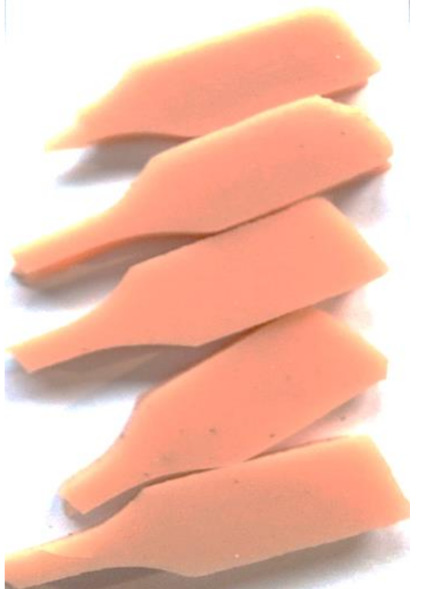
GCW10	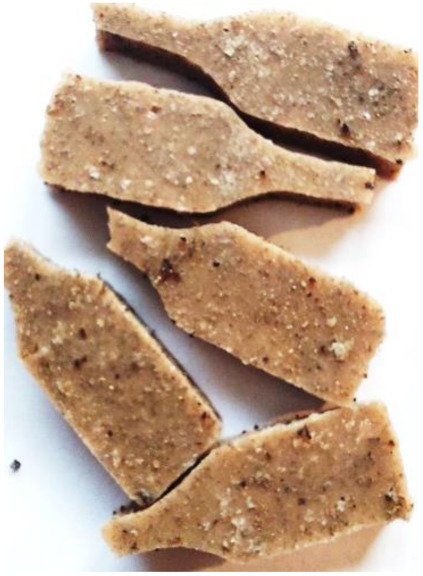	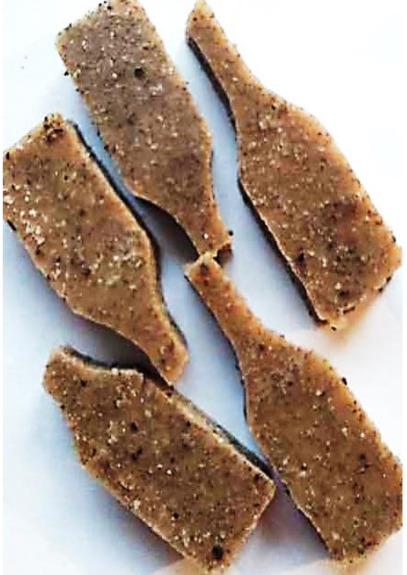	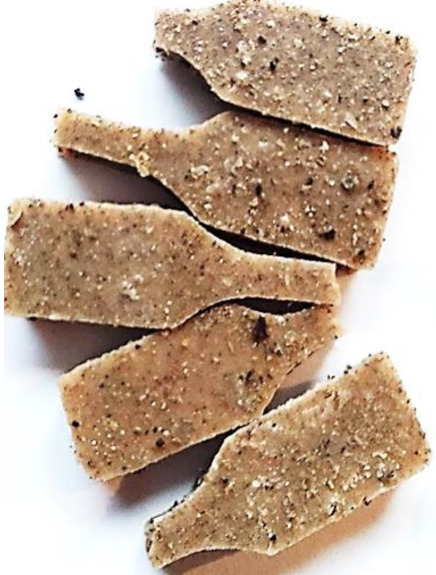
GCW20	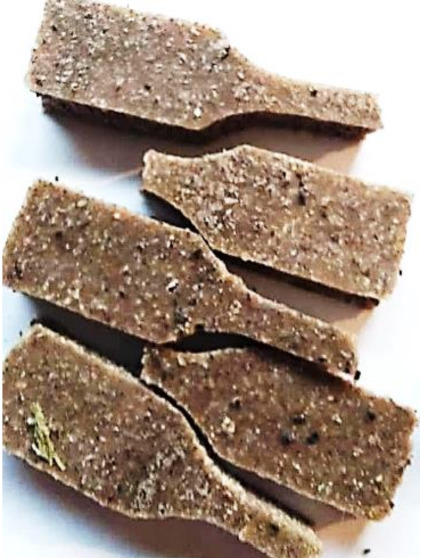	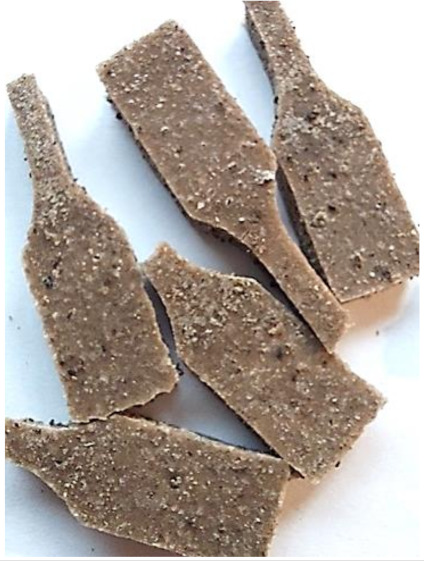	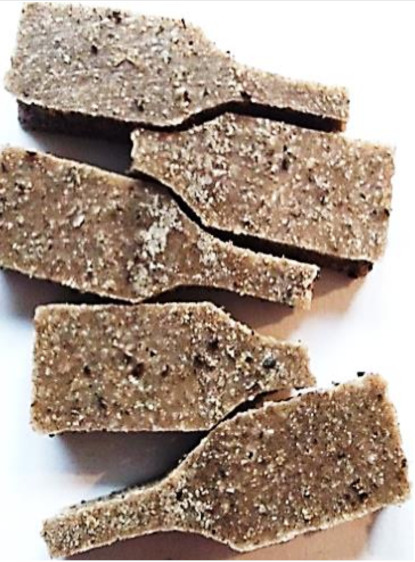
WS10	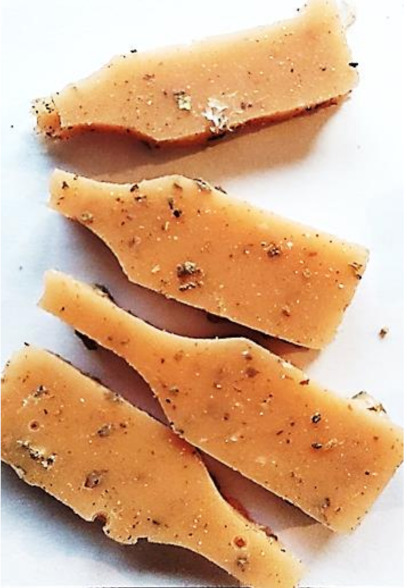	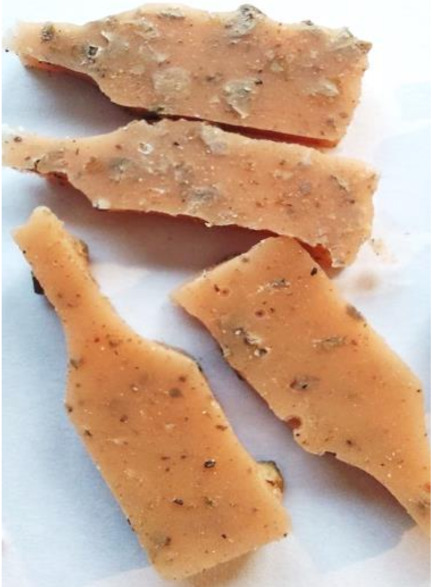	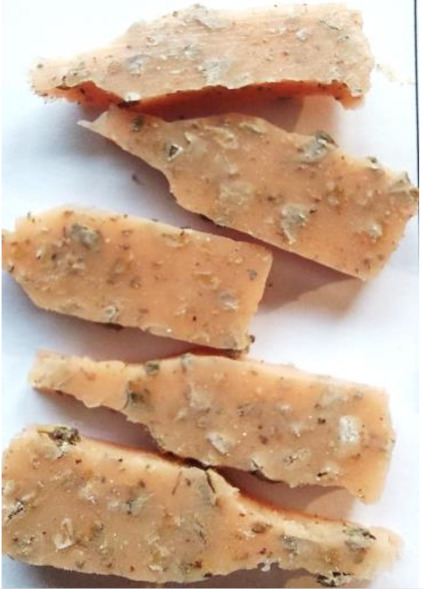
WS20	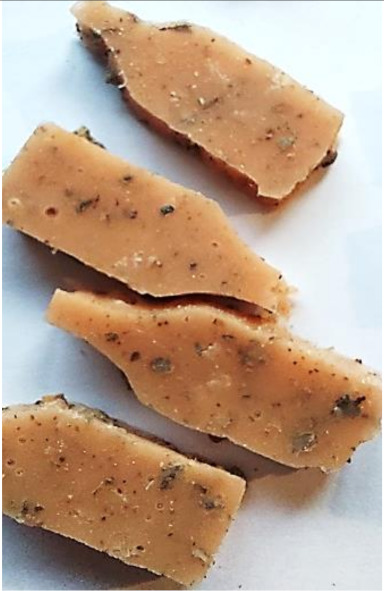	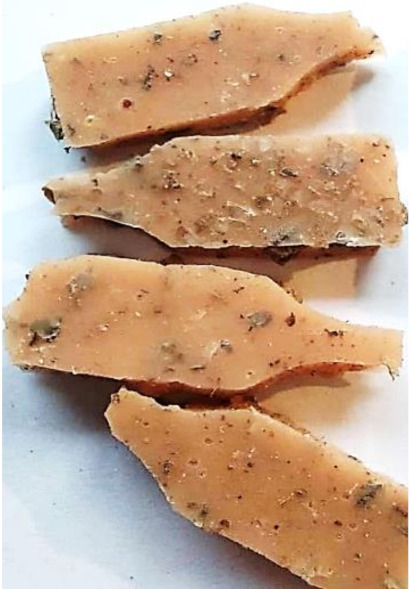	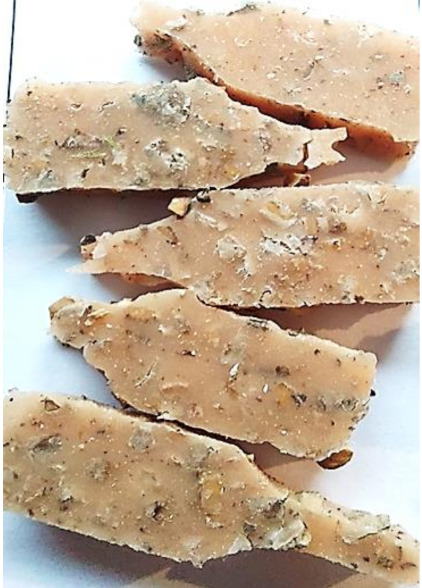
BSG10	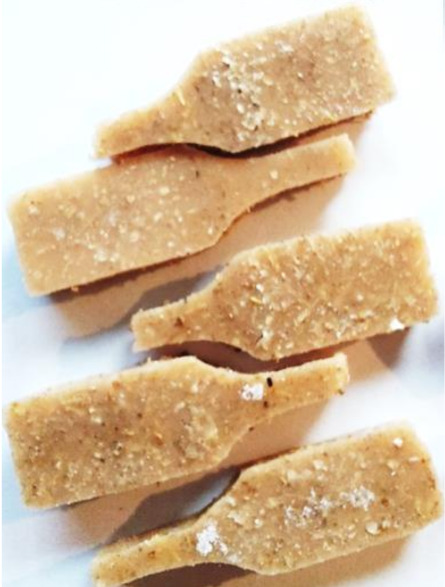	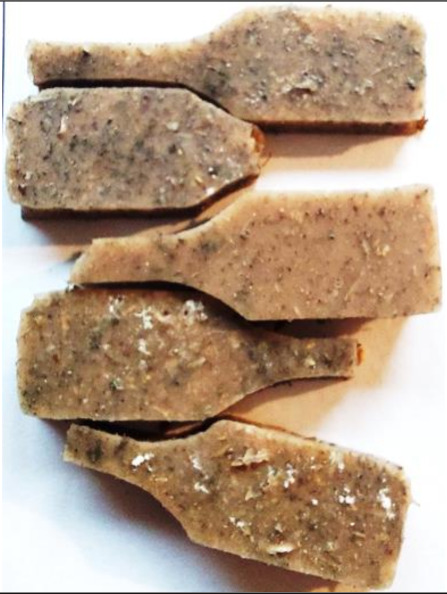	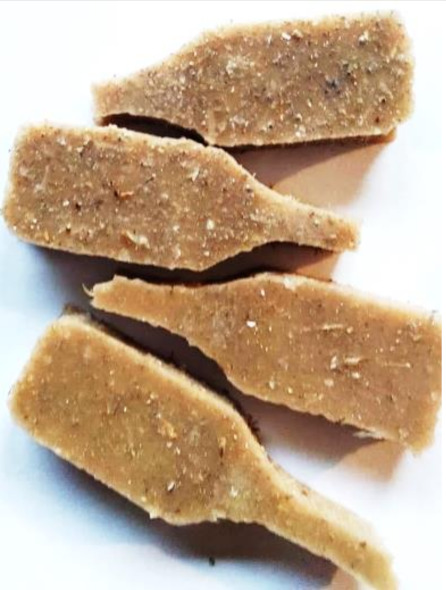
BSG20	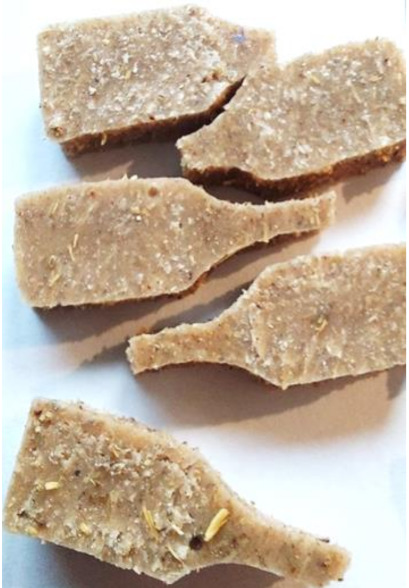	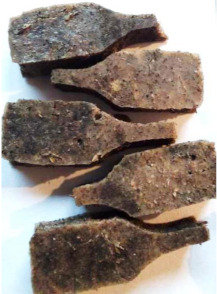	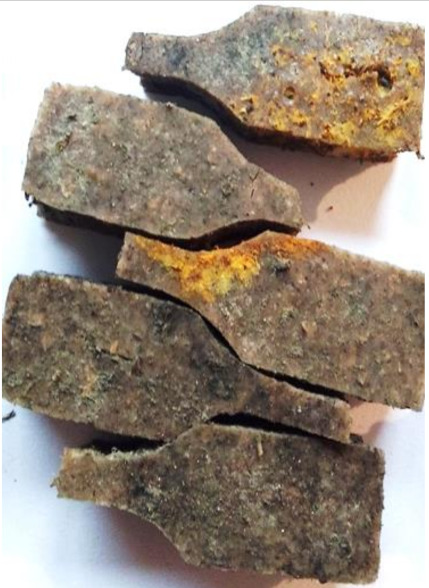
PS10	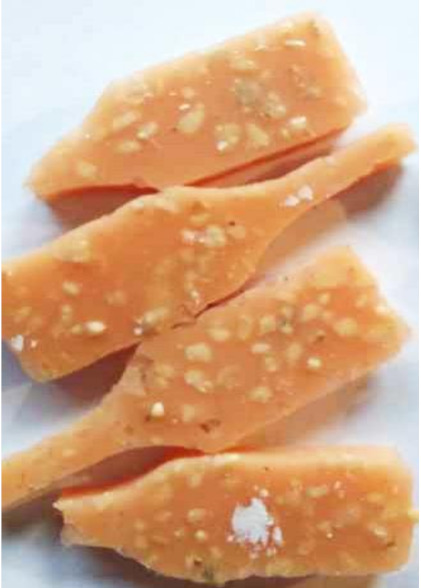	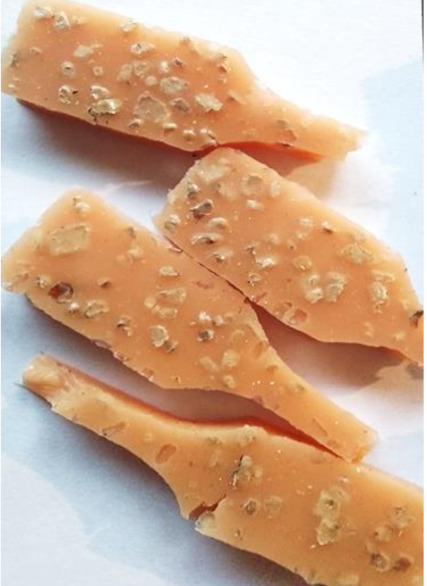	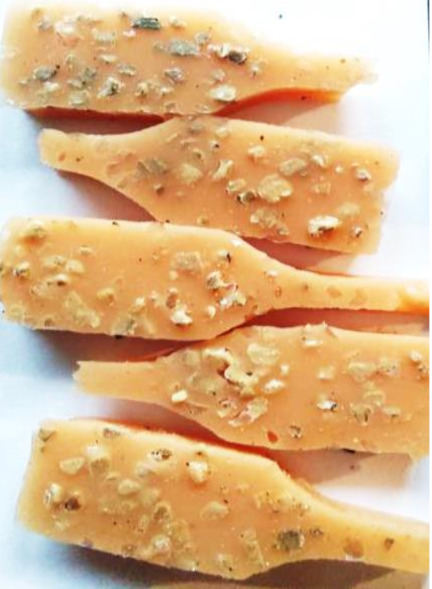
PS20	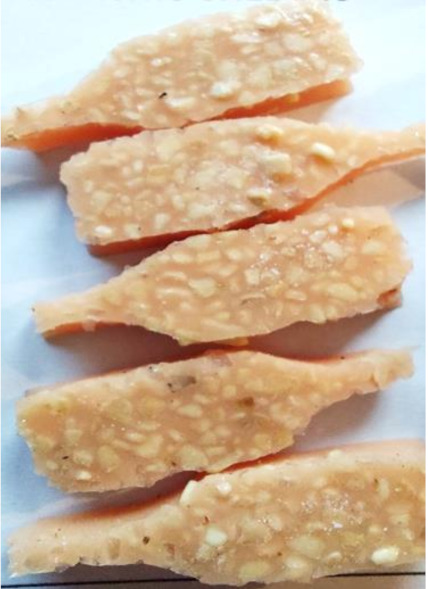	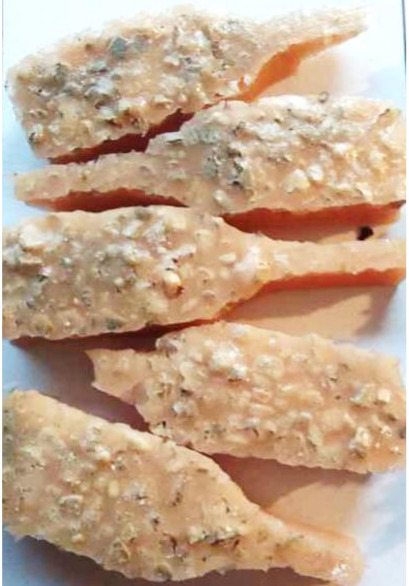	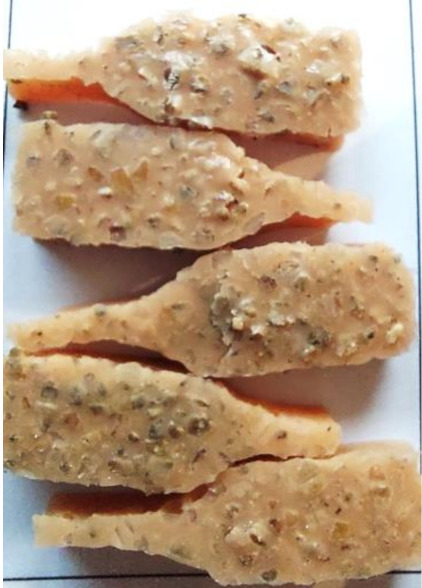
CH10	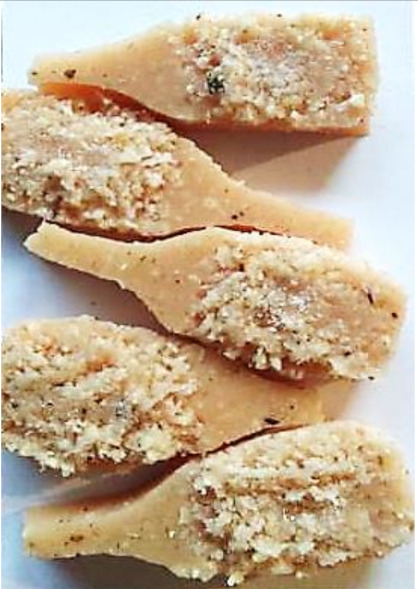	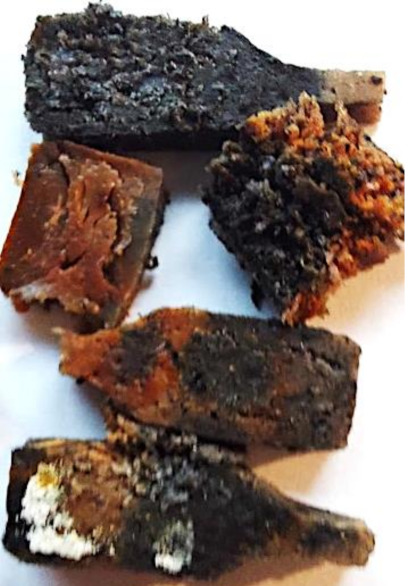	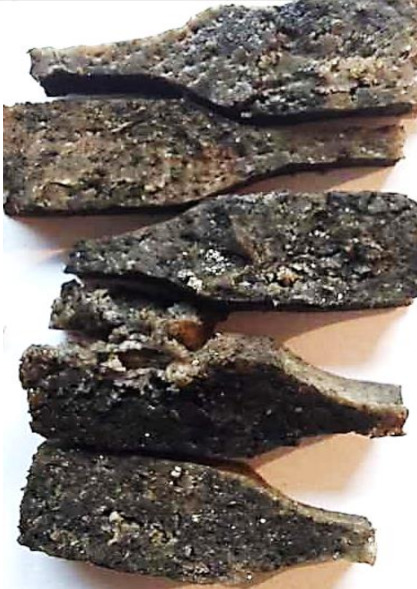
CH20